# Increase of nesting habitat suitability for green turtles in a warming Mediterranean Sea

**DOI:** 10.1038/s41598-023-46958-4

**Published:** 2023-12-07

**Authors:** Chiara Mancino, Sandra Hochscheid, Luigi Maiorano

**Affiliations:** 1 Department of Biology and Biotechnologies ‘‘Charles Darwin’’, Sapienza University of Rome, Viale Dell’Università 32, 00185 Rome, Italy; 2 Marine Turtle Research Group, Department of Marine Animal Conservation and Public Engagement, Stazione Zoologica Anton Dohrn, Napoli, Italy

**Keywords:** Marine biology, Ecology, Zoology, Ecology

## Abstract

Climate change is reshaping global ecosystems at an unprecedented rate, with major impacts on biodiversity. Therefore, understanding how organisms can withstand change is key to identify priority conservation objectives. Marine ectotherms are being extremely impacted because their biology and phenology are directly related to temperature. Among these species, sea turtles are particularly problematic because they roam over both marine and terrestrial habitats throughout their life cycles. Focusing on green turtles (*Chelonia mydas*) in the Mediterranean Sea, we investigated the future potential changes of nesting grounds through time, assuming that marine turtles would shift their nesting locations. We modeled the current distribution of nesting grounds including both terrestrial and marine variables, and we projected the potential nesting distribution across the Mediterranean basin under alternative future greenhouse gas emission scenario (2000–2100). Our models show an increase in nesting probability in the western Mediterranean Sea, irrespective of the climate scenario we consider. Contrary to what is found in most global change studies, the worse the climate change scenario, the more suitable areas for green turtles will potentially increase. The most important predictors were anthropogenic variables, which negatively affect nesting probability, and sea surface temperature, positively linked to nesting probability, up to a maximum of 24–25 °C. The importance of the western Mediterranean beaches as potential nesting areas for sea turtles in the near future clearly call for a proactive conservation and management effort, focusing on monitoring actions (to document the potential range expansion) and threat detection.

## Introduction

Anthropogenically induced climate change is quickly reshaping the world’s ecosystems with major impacts on biodiversity^1^. Marine ecosystems have been particularly hit, with roughly 66% of the global oceans being affected by multiple human pressures^2^. The consequences for marine biodiversity are severe. The Ocean Living Planet Index indicates that global ocean fish stocks have been overexploited by 29%, ocean species have decreased by 39%, and world coral reefs have decreased by 50%^3^. Among all threats affecting marine ecosystems, climate change is currently considered one of the most important^4^, with 14% of all marine species already heavily impacted by climatic factors^5^ and with increasingly severe impacts over time. The extreme heat waves in 2019 hit 57% of the ocean surface waters, with significant impacts on marine ecosystems and their ability to provide resources to coastal communities^6^.

Future projections return an overly grim outcast, even considering the near future, with the Mediterranean Sea representing one of the most vulnerable regions in the world when considering the impacts of global warming^7^. The Mediterranean has always been hugely exploited by humans, with important and potentially negative interactions between human activities and climate changes. The basin currently hosts more than 500 million inhabitants, a third of whom live along the coasts, and is the first tourist destination in the world with 360 million visitors per year^8,9^. The combination of climate change and human impacts clearly generate the starting baseline for a biodiversity collapse^10^, especially for species exploiting at the same time marine and terrestrial habitats as sea turtles.

Many species of vertebrates (birds, mammals, and reptiles) roam over both the marine and terrestrial realm and are potentially very sensitive to global changes. In fact, they exploit very different habitats during their life cycles, often migrating over long distances to reach nesting, feeding, and reproductive grounds^11,12^, being therefore exposed to multiple and often uncoupled threats. Many of these species (e.g., *Mirounga leonina*, *Aptenodytes patagonicus*) are responding to climate change by changing their phenology and/or distribution range^13,14^, while others (e.g., *Fratercula arctica*) seem unable to adapt fast enough^11^.

Single species responses are expected to lead to changes also in the pattern of species richness^15^, in community and ecosystem structure^16^, and in ecosystem services^17^. Although negative impacts are predominant, climate changes could also have positive effects on marine biodiversity. For instance, increased temperatures and CO^2^ concentrations seem to accelerate primary biomass production^18^, while milder winters might increase survival probabilities of many threatened species in temperate regions^19,20^.

Understanding the impact of global changes in marine systems is particularly challenging given their complexity and the huge number of factors that should be considered. Umbrella and/or keystone species can clearly represent a good proxy for global change effects, and marine turtles are particularly interesting in this context. In fact, during their life cycles, they exploit different habitats, nesting on the terrestrial realm and then using both the neritic and oceanic habitats, being potentially exposed to multiple, unrelated, and very different threats^11^. Being highly mobile and migratory species, they have the potential to adapt to changing conditions^21^ and can respond to changes by shifting their spatiotemporal distribution and/or changing their nesting and foraging behaviour^22,23^. However, sea turtles have a peculiar reproductive physiology, and therefore are particularly sensitive to climatic conditions during eggs incubation and development^24^, when climate change can directly affect sex ratio in hatchlings^25^. In fact, turtles have temperature-dependent sex determination, with females associated with high incubation temperatures (roughly above 29 °C)^26^ and males associated with lower temperatures, but extreme hot temperatures of the nests, ranged from 28.4 to 33.5 °C, can lead to decreased hatchling rates or even to complete nest failures^27–29^. Some authors have proposed a positive effect of increasing temperatures on sea turtle populations, suggesting that more female hatchlings would automatically translate into more breeding females, more nests and, consequently, population growth^30,31^. However, an excessive shortage of males can produce negative consequences (e.g., reductions in egg fertility, which may threaten population viability)^32^ and extreme temperatures may even lead to embryonic mortality^33^. Climate changes can also impact adult turtles. Being ectothermic, seawater temperature can radically impact physiology and behaviour of turtles^34^. In fact, below a thermal threshold of around 10 − 15 °C (depending on the species) their metabolic rates decrease, and turtles become less mobile^35–37^. On the other side, higher temperatures can induce indirect stress by promoting the growth of pathogens, with clear consequences on the biology of populations^38^. Overall, different analyses have predicted significant losses in nesting habitat for sea turtle populations in the Great Barrier Reef, Greece, and the Hawaiian Islands, even exceeding 65% of the areas suitable for nesting^39–42^. However, these projections, catastrophic in some cases, are often focused exclusively on climate and do not consider other important factors which can contribute to an even worse outcome. For example, although the relationship between climate change and sea level rise and coastal storms in the Mediterranean Sea is very weak^43^, the flooding of beaches, associated with an increasing prevalence and intensity of storms and sea level rise, will further reduce both hatching success and availability of nesting habitats^27,44^.

Currently, most research efforts in relation to climate change impacts in the Mediterranean have been related to loggerhead sea turtles (*Caretta caretta*), focusing on the current and potential distribution^23^, ecology^45^, and conservation of the species^46^. Much less is known about green turtles (*Chelonia mydas*), for which only a general overview of the ecology and conservation status exists^47^. A recent analysis^48^, considering only bioclimatic variables, found no appreciable change in the potential suitability for green turtle nesting grounds under different global change scenarios in the Mediterranean. As a species tightly linked to sea surface temperature, with an optimum temperature range for functioning > 25 °C^49–53^, these results are surprising. In fact, green turtles are strongly adapted to hot conditions with a distribution that is mostly tropical. In the Mediterranean, they are limited to the hottest areas, and particularly to the Levantine basin (Turkey, Syria, Cyprus, Lebanon, Israel, Egypt). Their nesting grounds are all in the easternmost part of the Mediterranean, with the vast majority of the nests located in Cyprus and Turkey^54^. Foraging areas extend to Greece and Libya^47^, while a few wandering green turtles can be occasionally found in the Adriatic Sea and very rarely in the western basin.

Using a species distribution modelling approach fully focused on the species ecology, and therefore including both marine and terrestrial variables, anthropogenic and natural factors, we provide here a thorough investigation of the impact of future climate change scenarios on the distribution of nesting grounds for green turtles in the Mediterranean basin. Our main hypothesis, contrary to what is available in the literature for the same species^48^, is that nesting ground availability for green turtles will potentially increase in the Mediterranean following hotter summers and winters, shifting their nesting locations over extended periods ranging from years to decades in response to climate change-induced warming and its associated impacts. If our hypothesis is confirmed, our results can be extremely important for a proactive management approach in which the conservation of the species and the management of potential conflicts with human coastal activities is planned and enforced well before the species’ arrival.

## Methods

### Nesting locations

We built a database on green turtles’ nesting locations searching Scopus, Web of Science, and Google Scholar with the following keywords: “sea turtle*” OR “marine turtle*” AND “nesting habitat” AND “Mediterranean”. We combined the list of references obtained from the 3 search engines and obtained a first set of 37,580 references. We also downloaded all documents referred in the first set of references and obtained a total of 38,590 peer reviewed papers and technical reports. We scanned all documents, and we retained only documents dealing with green turtles in the Mediterranean, looking for indications on nest location, excluding all dubious observations (nest attempted, nest possible but not proved, false crawls) and duplicate records (same date and same beach reported in different documents).

### Environmental data and scenarios

To model the potential distribution for nesting sites we considered environmental, anthropogenic, and climatic variables spanning both the marine and the terrestrial realm. We considered a total of 14 variables directly linked to nesting ecology in green turtles and summarized each variable over May–August to specifically include the nesting season^55^ (Table 1).

**Table 1:** Environmental and climatic variables included (✓) or excluded (X) in the final model.

Environmental and climatic variables	Final set of variables	References
Sea surface temperature (sst) [°C]	✓	^56^
Practical salinity units (psu) [psu]	✓	^56^
Concentration of chlorophyll in sea water (chl) [mg/m^3^]	✓	^57^
Concentration of dissolved oxygen in seawater (O_2_) [mmol/m^3^]	✓	^57^
Concentration of nitrate in seawater (NO_3_) [mmol/m^3^]	✓	^57^
Oceanic pH (pH) [pH]	✓	^57^
Bathymetry (bathy) [m]	✓	^58^
Sandy coastlines (beach) [m]	✓	^59^
Human population (pop) [number of persons/km^2^]	✓	^60^
Artificial night sky brightness (light) [mcd/m^2^]	✓	^61^
Partial pressure of carbon dioxide (pCO_2_) [Pa]	X	^57^
Net primary production in sea ​​(pp) [mol/m^3^/s]	X	^57^
Concentration of phytoplankton in seawater (phyc) [mmol/m^3^]	X	^57^
Concentration of phosphate in seawater (PO_4_) [mmol/m^3^]	X	^57^

We considered sea surface temperature and salinity because green turtles show a strong affinity to areas with warmer sea temperatures in the Mediterranean Sea, thriving at SST of roughly 27–29 °C and with highly variable salinity (range 1.2–35.5‰)^49,51–53^. Furthermore, the spatial distribution of adults is closely tied to sea surface temperature^62,63^, which can influence their selection of nesting sites^64,65^. We also considered several variables related to water chemistry that may positively and/or negatively influence nesting site selection^66,67^. Excessive concentrations of nitrogen, often used in agriculture and spilled in sea waters, cause water pollution, eutrophication, and acidification^68^, adversely affecting sea turtle livelihoods, foraging, and nesting habitats^69^. Dissolved oxygen concentration, on the contrary, was chosen as a parameter to determine the health of water bodies^70^. Poor water quality can negatively impact sea turtle health, altering their distribution^71^. On land, green turtle nests are clearly related to the presence of sandy beaches but are adversely affected by urbanization and other types of anthropogenic developments which usually besets sandy shores^72^. In fact, green turtles are often forced to use sub-optimal nesting habitats, competing for their nests with tourists and coastal residents^73,74^.

To limit the negative effects of multicollinearity on model calibration^75^, we performed a Variance Inflation Factor analysis (VIF) on the initial set of 14 variables and we excluded all variables with a VIF ≥ 3 obtaining a final database with 10 variables (Table 1).

We downloaded all available future layers at 2100 for the same variables from Bio-Oracle ^76,77^ (~ 9.2 km resolution) and from the SEDAC (Socioeconomic Data and Applications Center) ^60^ (1 km resolution). For future projections, we considered the following RCP (Representative Concentration Pathway) from CMIP-5 (Coupled Model Intercomparison Project): RCP2.6, a peak-and-decline scenario ending in very low greenhouse gas concentration levels by the end of the twenty-first century; RCP4.5 and RCP6.0, both with greenhouse gas concentrations stabilizing; and RCP8.5, a scenario of increasing emissions over time, leading to high greenhouse gas concentration levels^77^.

Green turtles are highly mobile animals, clearly influenced in their movements and in their ecology by environmental conditions occurring over large areas. Therefore, we applied to each variable considered in the model a moving window function in ArcGIS pro (ESRI ©) to summarize in each pixel the environmental characteristics of the neighbouring areas. For marine variables we considered a 20 km radius window to account for the mobility of the animals that typically swim within a mean distance of 20 km from beaches during nesting season^78,79^. For terrestrial variables we chose a 2 km radius to account for the limited movements when the animals are close to the shore (± 2 km)^80^. For all variables, the focal function assigned to the central pixel of the moving window the mean value calculated over all pixels inside the window.

### Modelling approach

To model the current species distribution of green turtles’ nesting grounds over the Mediterranean shores we used an information theory approach^81^. We calibrated the model using a maximum entropy algorithm (Maxent v. 3.4.1)^82^ considering all available nesting locations and 10,000 random background points sampled along the coasts of the eastern Mediterranean Sea (Levantine Sea, Tunisian Plateau, and Aegean Sea)^83^. We tuned model parameters according to Muscarella et al.^84^, with regularization multiplier ranging from 0.5 to 5.0 (0.5 increments) and with five different combination of feature classes: linear only, linear and quadratic; linear, quadratic, and hinge; linear, quadratic, hinge, and product; linear, quadratic, hinge, product, and threshold. Based on Li et al.^85^, we estimated the corrected Akaike Information Criteria (AICc) for each combination of feature classes and regularization multipliers. Variable contribution for each explanatory variable was evaluated using a jackknife approach^86^.

We evaluated the predictive capacity of the final model using a split-plot approach: we randomly split the set of occurrences and background points and we used 80% of the occurrences/background to calibrate a model and the remnant 20% for evaluation. We repeated the same process for 10 replicates measuring the AUC value (Area Under the receiver operating characteristic Curve). We obtained the average and standard deviation AUC and tested the statistical significance^87^.

There are several ways to correct sampling bias. Spatial filtering may not be helpful when there are only a few presence points^88^. For this reason, we utilized clumping, which reduces the training sample size. Depending on the heterogeneity of the surrounding environment and the selected spatial resolution, it determines which areas in the climate landscape fall outside the range represented by the training data^88^.

We projected the final model over the entire coastline of the Mediterranean Sea both under current conditions and future scenarios. We converted the continuous models into binary predictions of presence and absence using a threshold corresponding to the 10th percentile of probability values measured over the occurrences, and we used the binary model to calculate the percentages of changes in species presences from current conditions to future scenarios for the entire Mediterranean Sea.

### Ethical approval

All methods were carried out in accordance with relevant guidelines and regulations.

## Results

**Figure 1:**
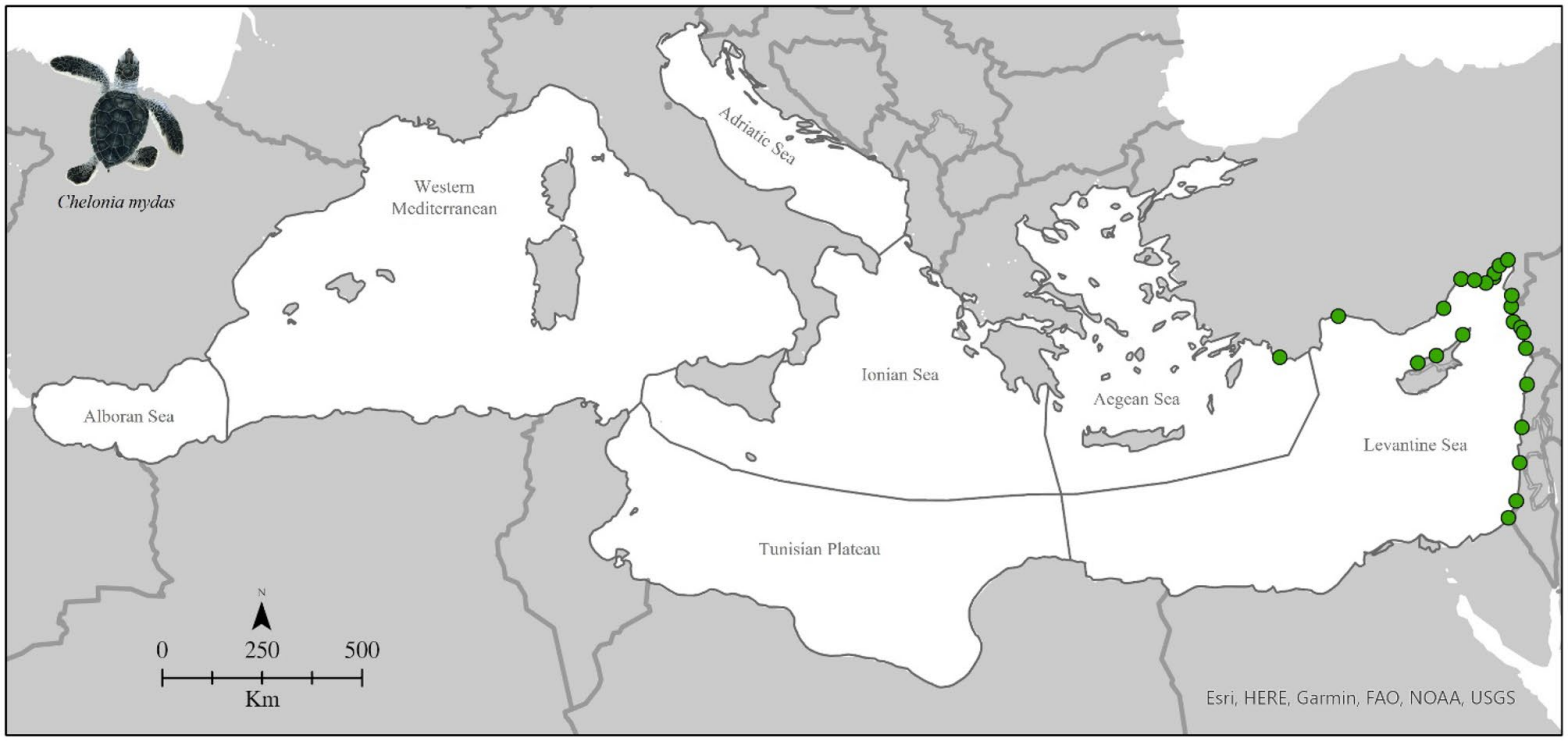
Nesting locations of *Chelonia mydas* in the Mediterranean basin from 1982 to 2019.

From the initial set of 38,590 references, only 23 reported information on green turtle nesting grounds, covering 5 countries, all in the eastern Mediterranean basin (Table 2). Overall, we obtained 178 nesting locations of green turtles going from 1982 to 2019, located from Turkey to the southern boundaries of Israel (Fig. 1). All nests were in the Levantine Sea ecoregion (Fig. 1), except a single nest in the Aegean Sea (but still in Turkey) collected in 2000.

**Table 2:** Number of locations for each country, with their references (complete list in Appendix A in Supplementary material, Table A.1).

Country	Number of locations	References
Turkey	106	Canbolat, (2004)
		Erdogan et al., (2001)
		Ergene et al., (2016)
		Kaska et al., (1998)
		Kasparek et al., (2001)
		Önder & Candan, (2016)
		Özdilek et al., (2016)
		Sönmez, (2018)
		Sönmez et al., (2021)
		Turan et al., (2021)
		Türkozan et al., (2023)
		Yalçın-Özdilek, (2007)
		Yılmaz et al., (2015)
		Yılmaz et al., (2022)
		Yılmaz & Oruç, (2022)
Cyprus	44	Broderick & Godley, (1996)
		Broderick et al., (2002)
		Hays et al., (2002)
		Hochscheid et al., (2018)
		Kasparek et al., (2001)
Israel	21	Kasparek et al., (2001)
		Mazor et al., (2013)
Syria	6	Kasparek, (1995)
		Rees et al., (2008)
Lebanon	1	Newbury et al., (2002)

### Current and future nesting model

The final potential nesting probability model (AICc values in Appendix A, Supplementary material, Table A.2) showed a very good predictive power with a mean AUC = 0.943 (± 0.020; *p* value ≤ 0.001). The average sea surface temperature, salinity, and human population density explained 98.8% of the variance of the entire model. Sea surface temperature was by far the most important variable, with salinity and human population density having a marginal effect on the potential distribution of nests.

**Figure 2:**
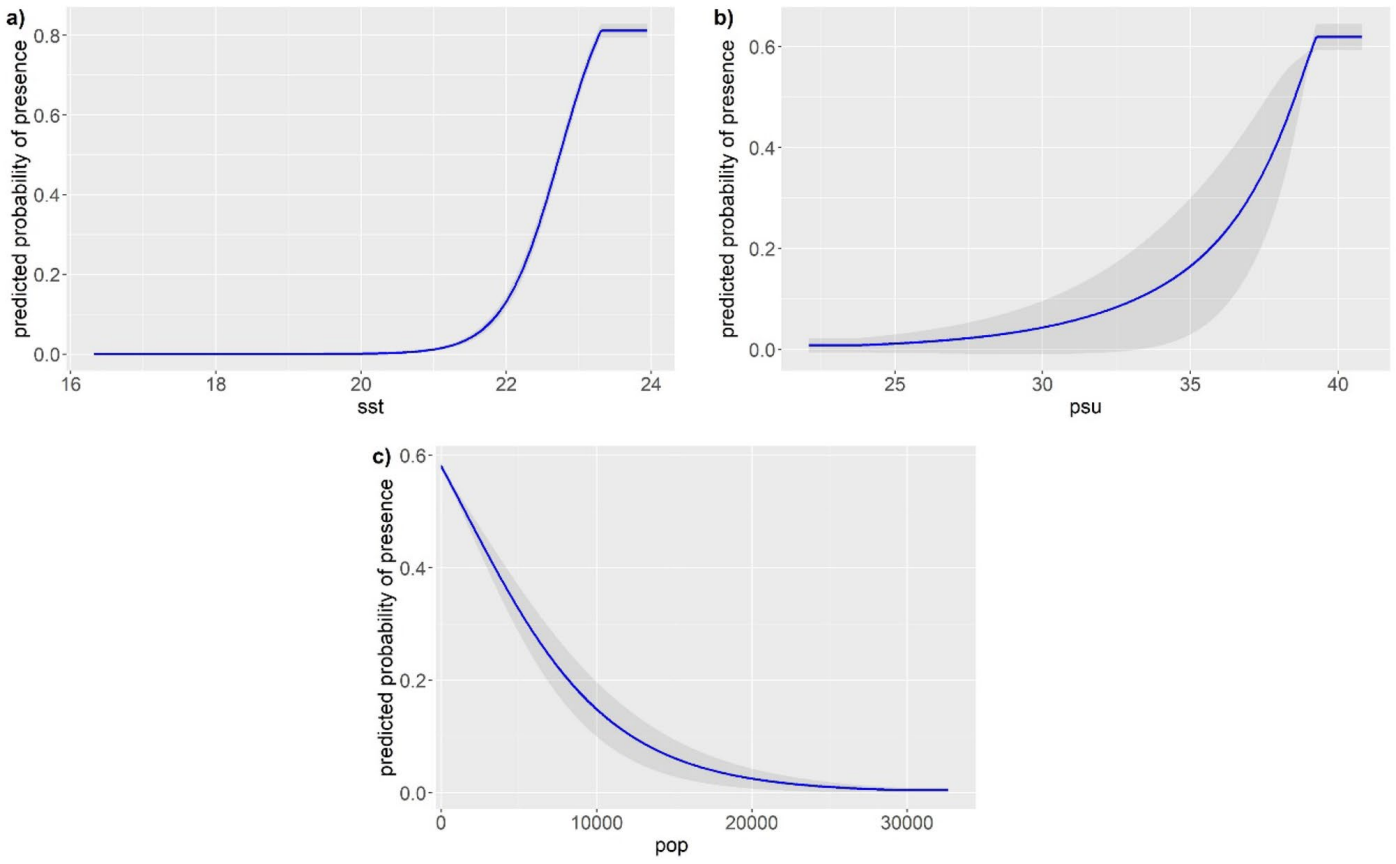
Marginal response curves for three variables: (**a**) sea surface temperature (sst), (**b**) salinity (psu), (**c**) human population density (pop). The curve represents the mean response to green turtle nesting probability, and the shaded areas represent one standard deviation both calculated over 10 replicates.

The probability of nesting increases with increasing sea surface temperatures and salinity (Fig. 2), being basically zero for temperatures below 21 °C and increasing up to a plateau corresponding to slightly more than 23 °C and 38‰ of salinity. On the contrary, the probability of nesting decreased with higher human population densities (Fig. 2).

**Figure 3:**
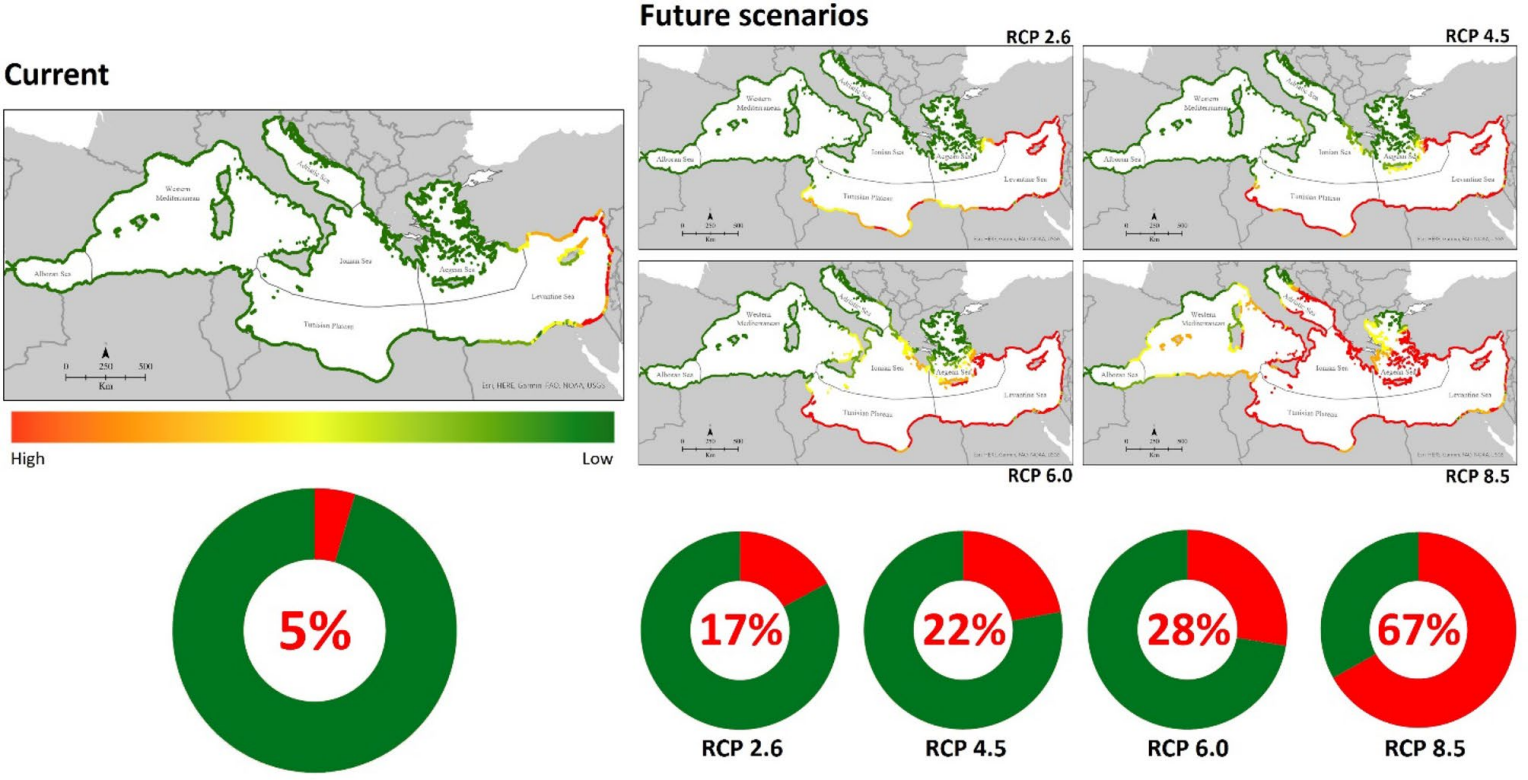
Distribution maps of environmental suitability for Chelonia mydas nesting habitat in the Mediterranean Sea from current, with only 5% of suitable areas, to future at 2100, with a 67% increase in the RCP scenario considering the highest levels of CO_2_ emissions. Percentages of change are calculated over the binary outputs (Appendix A in Supplementary material, Table A.3).

Considering current climate, the highest probabilities of nesting are limited to the eastern Mediterranean basin, and particularly to the southern shores of Turkey, to Cyprus, the middle East, and to the easternmost coasts of Egypt. Overall, only 5% of the Mediterranean shores are currently suitable as nesting grounds for the green turtle (Fig. 3). Whatever future scenario we consider, suitability for nesting will increase responding positively to increasing temperatures. Considering RCP2.6, RCP4.5, and RCP6.0 at 2100 the increase in suitability will be limited to the coasts of Turkey, Middle East, and North Africa, going westward up to the southern Tunisia (Fig. 3). According to RCP8.5, suitability will increase to cover 67% of the Mediterranean basin, with the north African coasts being almost completely suitable (up to Algeria), and with the Italian coasts representing good habitat for the species (Fig. 3).

## Discussion

Overall, in the vast majority of biological systems the impacts of ongoing and future climate changes are driving towards a huge loss of biodiversity^89,90^, with consequences also for human wellbeing^91^. Only a limited number of studies highlight a potentially positive effect, for example the increase in average carbon dioxide levels over the past century positively affected plant growth rates^92,93^ and primary biomass production^18^. In this study we have shown that future climate change can potentially lead to an increase in the nesting distribution of green turtles, which theoretically should expand their nesting ground towards the western Mediterranean Sea.

Green turtles have a global distribution that spans primarily the tropical, subtropical, and temperate regions of the Atlantic, Pacific, and Indian Oceans, with a small population also in the Mediterranean Sea^66,94^. Here, their nesting grounds have always been limited to the Levantine basin, with 99% of nesting records limited to Turkey and Cyprus, and with occasional nesting along the shores of Egypt, Israel, and Lebanon^95^. During the first half of the twentieth century, the Mediterranean population of green turtles have declined dramatically due to loss or deterioration of nesting beaches, adverse pressure from incidental catch in marine fisheries (particularly trawling), and marine pollution^96^. Most conservation efforts have been concentrated on creating protected areas to safeguard nesting beaches, controlling beach nourishment, and minimizing human influences. All these efforts have certainly contributed to a sharp reduction in these problems, and monitoring data now show an increase in nesting activities and a higher number of mature females in the Mediterranean^97^. These changes are still limited to the eastern Mediterranean basin, but vagrant individuals are constantly seen in the western Mediterranean in the last few years^98,99^.

The potential distribution we obtained with our model confirms that the current distribution of nesting grounds for the green turtle is limited to the easternmost part of the Mediterranean basin, the only part where SST is optimal^100^. The model closely follows existing nests, with only marginal suitability along the Egyptian shores where no nest has been recorded. We found a very narrow interval of sea surface temperatures that defines a beach as suitable for nesting, with optimal nesting grounds corresponding to a SST of 25 °C in front of the shore. This value corresponds strikingly well with the results obtained from empirical field studies, which suggests that female turtles during the nesting period actively select waters with temperatures > 25 °C^49–53^.

In our model's future projections suitable areas increase in the western basin, and the temperature increase reaches a plateau while still remaining below 26 °C. Since 1978 the trends in SST in the Mediterranean Sea increased by 1.4 °C^101,102^, and the basin is constantly warming by 0.35 °C per decade, especially in spring by 0.38 °C, followed by 0.32 °C in summer^103^, mostly in the Eastern Mediterranean^104^. All future scenarios project an increasingly hot Mediterranean Sea by the end of the twenty-first century, ranging from an average annual increase of 0.45 °C for the RCP2.6 scenario to 2.56 °C for the RCP8.5 scenario. In each scenario, the summer, a key season for sea turtles, displayed the maximum warning trend^103^. Our model will continue to predict high suitability even if temperatures are excessively high. Therefore, we recommend that in situations with a strong sampling bias towards some regions or environmental features, caution should be exercised to avoid over-interpreting the results. Additionally, it is advisable to reduce spatial clumping of records in datasets used for Maxent model calibration^105^.

Contrary to Arslan et al.^48^, who found no significant differences in the suitability of nesting areas for green turtles from the past to the future, we found that nesting habitat for the green turtle in the Mediterranean will increase in the future irrespective of the climate scenario we considered, and the increase in suitability is going to be stronger for more extreme scenarios. This discrepancy could possibly result from the different explanatory variables considered. However, it’s very important to consider an information theoretic approach or another type of variable reduction approach otherwise overfitting problems could easily drive the modelling results^106^, leading to a model that fit random noise in the data^107^ instead of ecological signals.

Clearly, the capacity of species to respond to environmental and climate changes depends on a combination of different elements, going from phenological plasticity to range shifts, and ultimately to rapid evolution of traits better suited to new conditions^108^. Moreover, marine turtles have persisted through dramatic climate change events during their past evolutionary history, clearly demonstrating the ability to adapt to changing conditions^64,109^. It is true, however, that current climate change is occurring at unprecedented speed^110^, questioning the evolutionary capacities of a k-selected species like the green turtle^111^. On the contrary, we do have empirical evidence that marine turtles, thanks to their high mobility, may respond to climatic changes by shifting their distribution range towards climatically suitable areas^23,112,113^. In fact, marine turtles may change the distribution of their nesting grounds, nest-site choice, and nest depth in the sand, adapting in situ by adjusting their pivotal temperature, and nesting in cooler months^114–118^. Moreover, sporadic nesting is thought to be a mechanism that enhances the ability of sea turtles to disperse and adapt to environmental changes, and new nesting populations can form in new regions where sporadic nesting is observed with increasing temperatures^119^. Indeed, to date more green turtle individuals have been sighted in the waters of the western Mediterranean, and the factor that has likely contributed to increased number of green turtles is rising sea temperatures^98,99^.

Range shifts may offer one of the most successful responses to climate change in marine organisms, and in marine turtles in particular^120^. However, especially considering human dominated landscapes like the Mediterranean basin, a high mobility may result in an increased exposure to threats, such as coastal development. In fact, around 40% of the total Mediterranean coastal zone are nowadays developed, with a projected increase with further coastal developments^121^. The human population in coastal areas grew from about 100 million in 1980 to 150 million in 2005. It could reach 200 million by 2030^8^. Coastal development, human presence, and associated pressures can modify marine turtle nesting environment and affect females ‘reproductive output’^122,123^. The direct and indirect impacts of coastal development on marine turtles can potentially reduce their ability to use and colonize new areas^124^. Driving on the beach and the use of heavy machinery for beach cleaning purposes are common practices and are responsible for alterations in sand characteristics and the destruction of turtle clutches^66^. Beach furniture, sports courts, and artificial lighting on some nesting beaches reduce the habitat available for nesting, prevent females from accessing suitable nesting sites and cause disorientation of hatchlings through light pollution^125^. In addition, people on the beach at night may disrupt nesting activity, causing the abandonment of nesting attempts or the destruction through trampling of incubating nests^125^.

In conclusion, our results show a potential expansion of *Chelonia mydas* in the western Mediterranean in response to climate change. Therefore, as soon as green turtle arrives in the west, management efforts should focus on enhancing marine turtle’s resilience to changing environmental conditions by mitigating other non-climatic threats they currently experience^112,126^. The choice of a particular beach as nesting ground it is also negatively influenced by urbanization and other types of development, which should be included in future studies. To better inform future management of marine turtles, there is the need to understand future threats, as well as to couple predictions of marine turtle distribution with an assessment of exposure to these threats^127^.

### Supplementary Information

Supplementary Information.

## Data Availability

Data will be made available on request to corresponding author: chiara.mancino@uniroma1.it.
