# Supplementary material for: Increase of nesting habitat suitability for green turtles in a warming Mediterranean Sea
Source: Sci Rep. 2023 Dec 7;13:19906. doi: 10.1038/s41598-023-46958-4 (PMC10703824; doi:10.1038/s41598-023-46958-4)
Supplement: Supplementary file 1 — Supplementary Information. [file 41598_2023_46958_MOESM1_ESM.docx]

**SUPPORTING INFORMATION**

**Appendix A**

| References |
| --- |
| Broderick. A. C.. & Godley. B. J. (1996). Population and nesting ecology of the green turtle. *Chelonia mydas*. and the loggerhead turtle. *Caretta caretta*. in Northern Cyprus. Zoology in the Middle East. 13(1). 27-46. |
| Broderick, A. C., Glen, F., Godley, B. J., & Hays, G. C. (2002). Estimating the number of green and loggerhead turtles nesting annually in the Mediterranean. Oryx, 36(3), 227-235. |
| Canbolat. A. F. (2004). A review of sea turtle nesting activity along the Mediterranean coast of Turkey. Biological Conservation. 116(1). 81-91. |
| Erdoğan. A.. Öz. M.. Kaska. Y.. Düşen. S.. Aslan. A.. Yavuz. M.. & Sert. H. (2001). Marine turtle nesting at Patara. Turkey. in 2000. Zoology in the Middle East. 24(1). 31-34. |
| Ergene. S.. Ergene. M.. Uçar. A. H.. Aymak. C.. & Kaçar. Y. (2016). Identification of a new nesting beach in Mersin. Turkey: Nesting activity of green and loggerhead sea turtles over 6 nesting seasons (2009-2014) at Davultepe Beach. Marine Turtle Newsletter. 149. 6-9. |
| Hays. G. C.. Broderick. A. C.. Glen. F.. Godley. B. J.. Houghton. J. D. R.. & Metcalfe. J. D. (2002). Water temperature and internesting intervals for loggerhead (*Caretta caretta*) and green (*Chelonia mydas*) sea turtles. Journal of Thermal Biology. 27(5). 429-432. |
| Hochscheid. S.. Kaska. Y.. & Panagopoulou. A. (2018). Sea Turtles in the Mediterranean Region: MTSG Annual Regional Report 2018. Draft Report of the IUCN-SSC Marine Turtle Specialist Group. 2018. |
| Kaska. Y.. Downie. R.. Tippett. R.. & Furness. R. W. (1998). Natural temperature regimes for loggerhead and green turtle nests in the eastern Mediterranean. Canadian Journal of Zoology. 76(4). 723-729. |
| Kasparek. M. (1995). The nesting of marine turtles on the coast of Syria. Zoology in the Middle East. 11(1). 51-62. |
| Kasparek. M.. Godley. B. J.. & Broderick. A. C. (2001). Nesting of the green turtle. Chelonia mydas. in the Mediterranean: a review of status and conservation needs. Zoology in the Middle East. 24(1). 45-74. |
| Mazor. T.. Levin. N.. Possingham. H. P.. Levy. Y.. Rocchini. D.. Richardson. A. J.. & Kark. S. (2013). Can satellite-based night lights be used for conservation? The case of nesting sea turtles in the Mediterranean. Biological Conservation. 159. 63-72. |
| Newbury. N.. Khalil. M.. & Venizelos. L. (2002). Population status and conservation of marine turtles at El-Mansouri. Lebanon. Zoology in the Middle East. 27(1). 47-60. |
| Önder. B. F.. & Candan. O. (2016). The feminizing effect of metabolic heating in Green Turtle (*Chelonia mydas*) clutches in the eastern Mediterranean. Zoology in the Middle East. 62(3). 239-246. |
| Özdilek. Ş. Y.. Sönmez. B. E. K. T. A. Ş.. & Kaska. Y. (2016). Sex ratio estimations of *Chelonia mydas* hatchlings at Samandağ Beach. Turkey. Turkish Journal of Zoology. 40(4). 552-560. |
| Rees. A. F.. Saad. A.. & Jony. M. (2008). Discovery of a regionally important green turtle *Chelonia mydas* rookery in Syria. Oryx. 42(3). 456-459. |
| Sönmez. B. (2018). Sixteen-year (2002-2017) record of sea turtle strandings on Samandağ Beach. the eastern Mediterranean coast of Turkey. Zoological Studies. 57. |
| Sönmez. B.. Karaman. S.. & Turkozan. O. (2021). Effect of predicted sea level rise scenarios on green turtle (*Chelonia mydas*) nesting. Journal of Experimental Marine Biology and Ecology. 541. 151572. |
| Turan. C.. Sönmez. B.. Gürlek. M.. DOĞDU. S.. Deniz. A. Y. A. S.. Ergenler. A.. & Türkmani. M. (2021). The Investigations of The Nesting Status of The Green Turtle *Chelonia mydas* on Yeniyurt Beach (Hatay) in The Northeastern Mediterranean. Turkey. Ecological Life Sciences. 16(1). 40-47. |
| Türkozan. O.. Yılmaz. C.. Almpanidou. V.. Godfrey. M. H.. & Mazaris. A. D. (2023). Thermal conditions of green turtle (*Chelonia mydas*) nests in the largest rookery in the eastern Mediterranean. Endangered Species Research. 50. 63-73. |
| Yalçın-Özdilek. Ş. (2007. January). Status of sea turtles (*Chelonia mydas* and *Caretta caretta*) on Samandağ Beach. Turkey: a five-year monitoring study. In Annales Zoologici Fennici (pp. 333-347). Finnish Zoological and Botanical Publishing Board. |
| Yılmaz. C.. Oruç. A.. & Türkozan. O. (2015). Marine turtles (*Chelonia mydas* and *Caretta caretta*) nesting along the eastern Mediterranean coast of Turkey: Results from six years of surveying. The Herpetological Journal. 25(4). 197-204. |
| Yılmaz. C.. & Oruç. A. (2022). Sex ratio estimation for Green Turtle. *Chelonia mydas*. hatchlings at Akyatan Beach. Turkey. Zoology in the Middle East. 68(4). 300-308. |
| Yılmaz. C.. Oruç. A.. & Turkozan. O. (2022). Abundance Trends and Nesting Biology of Green Turtles *Chelonia mydas* (Testudines: Cheloniidae) During Ten Consecutive Breeding Seasons (2012–2021) at Akyatan Beach. Turkey. Zoological Studies. 61. |

Table A.1. List of references of *Chelonia mydas* nesting site data collected from 1982 to 2019 in the Mediterranean Sea.

| Model number | Features | Regularization multiplier | AICc |
| --- | --- | --- | --- |
| 1 | linear (L) | 0.5 | 155.5 |
| 11 | linear (L) + quadratic (Q) | 0.5 | 159.1 |
| 21 | linear (L) + quadratic (Q) + hinge (H) | 0.5 | 159.1 |
| 31 | linear (L) + quadratic (Q) + hinge (H) + product (P) | 0.5 | 159.1 |
| 41 | linear (L) + quadratic (Q) + hinge (H) + product (P) + threshold (T) | 0.5 | 159.1 |
| 2 | linear (L) | 1 | 165.2 |
| 12 | linear (L) + quadratic (Q) | 1 | 166.0 |
| 22 | linear (L) + quadratic (Q) + hinge (H) | 1 | 166.0 |
| 32 | linear (L) + quadratic (Q) + hinge (H) + product (P) | 1 | 166.0 |
| 42 | linear (L) + quadratic (Q) + hinge (H) + product (P) + threshold (T) | 1 | 166.0 |
| 3 | linear (L) | 1.5 | 173.4 |
| 13 | linear (L) + quadratic (Q) | 1.5 | 173.6 |
| 23 | linear (L) + quadratic (Q) + hinge (H) | 1.5 | 173.6 |
| 33 | linear (L) + quadratic (Q) + hinge (H) + product (P) | 1.5 | 173.6 |
| 43 | linear (L) + quadratic (Q) + hinge (H) + product (P) + threshold (T) | 1.5 | 173.6 |
| 4 | linear (L) | 2 | 176.5 |
| 14 | linear (L) + quadratic (Q) | 2 | 177.6 |
| 24 | linear (L) + quadratic (Q) + hinge (H) | 2 | 177.6 |
| 34 | linear (L) + quadratic (Q) + hinge (H) + product (P) | 2 | 177.6 |
| 44 | linear (L) + quadratic (Q) + hinge (H) + product (P) + threshold (T) | 2 | 177.6 |
| 16 | linear (L) + quadratic (Q) | 3 | 178.6 |
| 26 | linear (L) + quadratic (Q) + hinge (H) | 3 | 178.6 |
| 36 | linear (L) + quadratic (Q) + hinge (H) + product (P) | 3 | 178.6 |
| 46 | linear (L) + quadratic (Q) + hinge (H) + product (P) + threshold (T) | 3 | 178.6 |
| 15 | linear (L) + quadratic (Q) | 2.5 | 178.8 |
| 25 | linear (L) + quadratic (Q) + hinge (H) | 2.5 | 178.8 |
| 35 | linear (L) + quadratic (Q) + hinge (H) + product (P) | 2.5 | 178.8 |
| 45 | linear (L) + quadratic (Q) + hinge (H) + product (P) + threshold (T) | 2.5 | 178.8 |
| 5 | linear (L) | 2.5 | 179.4 |
| 17 | linear (L) + quadratic (Q) | 3.5 | 180.9 |
| 27 | linear (L) + quadratic (Q) + hinge (H) | 3.5 | 180.9 |
| 37 | linear (L) + quadratic (Q) + hinge (H) + product (P) | 3.5 | 180.9 |
| 47 | linear (L) + quadratic (Q) + hinge (H) + product (P) + threshold (T) | 3.5 | 180.9 |
| 6 | linear (L) | 3 | 182.2 |
| 18 | linear (L) + quadratic (Q) | 4 | 183.0 |
| 28 | linear (L) + quadratic (Q) + hinge (H) | 4 | 183.0 |
| 38 | linear (L) + quadratic (Q) + hinge (H) + product (P) | 4 | 183.0 |
| 48 | linear (L) + quadratic (Q) + hinge (H) + product (P) + threshold (T) | 4 | 183.0 |
| 7 | linear (L) | 3.5 | 184.9 |
| 19 | linear (L) + quadratic (Q) | 4.5 | 185.1 |
| 29 | linear (L) + quadratic (Q) + hinge (H) | 4.5 | 185.1 |
| 39 | linear (L) + quadratic (Q) + hinge (H) + product (P) | 4.5 | 185.1 |
| 49 | linear (L) + quadratic (Q) + hinge (H) + product (P) + threshold (T) | 4.5 | 185.1 |
| 20 | linear (L) + quadratic (Q) | 5 | 187.2 |
| 30 | linear (L) + quadratic (Q) + hinge (H) | 5 | 187.2 |
| 40 | linear (L) + quadratic (Q) + hinge (H) + product (P) | 5 | 187.2 |
| 50 | linear (L) + quadratic (Q) + hinge (H) + product (P) + threshold (T) | 5 | 187.2 |
| 8 | linear (L) | 4 | 187.5 |
| 9 | linear (L) | 4.5 | 190.1 |
| 10 | linear (L) | 5 | 192.7 |

Table A.2. AICc values for the 50 models calibrated with diﬀerent combinations of feature classes and regularization multiplier values.

|  |  | **Not suitability areas** | **Suitability areas** | **% low suitability** | **% high suitability** |
| --- | --- | --- | --- | --- | --- |
| **Current** |  | 672143 | 32427 | 95.3 | 4.6 |
| **Future scenarios** | **RCP 2.6** | 584994 | 120506 | 82.9 | 17.0 |
|  | **RCP 4.5** | 550103 | 155397 | 77.9 | 22.0 |
|  | **RCP 6.0** | 510882 | 194618 | 72.4 | 27.5 |
|  | **RCP 8.5** | 233997 | 471503 | 33.1 | 66.8 |

Table A.3. Km of beaches suitable for nesting under current climate and alternative future scenarios.
